# Identifying facilitators and barriers to the uptake of medication for opioid use disorder in Washington, DC: A community-engaged concept mapping approach

**DOI:** 10.1371/journal.pone.0306931

**Published:** 2024-07-19

**Authors:** Britta Gullahorn, Irene Kuo, Artius M. Robinson, Johnny Bailey, Jennifer Loken, Tamara Taggart

**Affiliations:** 1 Department of Prevention and Community Health, George Washington University Milken Institute School of Public of Health, Washington, DC, United States of America; 2 Department of Epidemiology, George Washington University Milken Institute School of Public of Health, Washington, DC, United States of America; 3 Family and Medical Counseling Services, Inc., Washington, DC, United States of America; 4 HIPS, Washington, DC, United States of America; 5 Whitman-Walker Health, Washington, DC, United States of America; 6 Department of Social and Behavioral Sciences, Yale School of Public Health, New Haven, CT, United States of America; Sao Paulo State University (UNESP), Botucatu Medical School, BRAZIL

## Abstract

**Introduction:**

Opioid overdose is a major public health challenge. We aimed to understand facilitators and barriers to engagement in medication for opioid use disorder (MOUD) among persons with OUD in Washington, DC.

**Methods:**

We used a cross-sectional mixed-methods concept mapping approach to explore MOUD engagement between 2021–2022. Community members at-large generated 70 unique statements in response to the focus prompt: “What makes medication for opioid use disorder like buprenorphine (also known as Suboxone or Subutex) difficult to start or keep using?” Persons with OUD (n = 23) and service providers (n = 34) sorted and rated these statements by theme and importance. Data were analyzed with multidimensional scaling and hierarchical cluster analysis, producing thematic cluster maps. Results were validated by our community advisory board.

**Results:**

Seven themes emerged in response to the focus prompt: availability and accessibility; hopelessness and fear; unmet basic needs; characteristics of treatment programs; understanding and awareness of treatment; personal motivations, attitudes, and beliefs; and easier to use drugs. “Availability and accessibility,” “hopelessness and fear,” and “basic needs not being met” were the top three identified barriers to MOUD among consumers and providers; however, the order of these priorities differed between consumers and providers. There was a notable lack of communication and programming to address misconceptions about MOUD’s efficacy, side effects, and cost. Stigma underscored many of the statements, showcasing its continued presence in clinical and social spaces.

**Conclusions:**

This study distinguishes itself from other research on MOUD delivery and barriers by centering on community members and their lived experiences. Findings emphasize the need to expand access to treatment, dismantle stigma associated with substance use and MOUD, and address underlying circumstances that contribute to the profound sense of hopelessness and fear among persons with OUD–all of which will require collective action from consumers, providers, and the public.

## Introduction

Opioid overdose remains a public health threat in the United States (US). From April 2020 to April 2021, drug overdose deaths increased by 28.5%, resulting in more than 100,000 deaths [1]. Approximately 81.9% of overdose deaths in 2021 were attributable to opioids, with illicitly manufactured fentanyl involved in 72.7% of deaths [2]. In Washington, DC (DC), a region with the second highest opioid-involved overdose death rate in the country, the majority of overdoses occur among Black males ages 40–69 years [2, 3]. These trends highlight the need to expand harm reduction and other initiatives to combat opioid overdose.

### Determinants of opioid use disorder (OUD) and barriers to treatment

A combination of individual, social, and structural factors fuels the opioid overdose crisis. Drug supply and overprescribing of opioids are often touted as key contributors, while the intersection of racism, poverty, issues of unstable housing or homelessness, trauma, access to culturally-appropriate care, social networks, isolation, and feelings of hopelessness—among other determinants—receive less attention from policymakers and program developers [4–6]. The criminalization of drug use, particularly among the Black community, and the perpetuation of fear and stigma in the media has historically slowed efforts to confront the epidemic [4].

Medication for opioid use disorder (MOUD)—such as buprenorphine, buprenorphine/naltrexone, and long-acting naltrexone in conjunction with counseling services—are gold standard, evidence-based methods of reducing overdose incidence and mortality [7]. A Maryland-based study found the risk of overdose death to be 82% lower among people with OUD when receiving MOUD versus the alternative of no engagement in MOUD [8]. Beyond a reduction in death rates, MOUD has additional benefits including higher odds of abstinence, employment, housing, and engagement in primary health care [9–13].

Nevertheless, perceived and real barriers on the patient and provider side have created an environment in which only one in four who need MOUD receive it [7, 14, 15]. Despite recent changes in US policy to increase the availability of MOUD, locating programs and providers capable of prescribing and administering MOUD is a challenge [16–19]. Moreover, lack of motivation and misinformation about MOUD (e.g., replacing one drug or addiction with another), paired with internalized, enacted, and anticipated stigma surrounding MOUD and substance use, often keep patients from engaging in MOUD [17, 20, 21]. Further, negative views of MOUD and a lack of provider training and clinical resources may inhibit the implementation of necessary care [21, 22].

### Community-based participatory research

Community-based participatory research (CBPR) is a process that engages and includes community members in all stages of program development to increase collaboration, gain community input, and increase program relevance [23–25]. The benefits of a CBPR approach are numerous, including: establishing mutual trust across community systems; gaining community perspectives in developing program components; gaining an understanding of community history, culture, and dynamics, and how program strategies used in other communities may or may not apply to the DC setting; and establishing relationships across communities to share resources and build research capacity. Given that the opioid epidemic in DC largely affects Black males ages 40–69 years, CBPR approaches may be particularly useful in understanding barriers and facilitators to accessing MOUD among this population which in turn can be used to develop strategies to increase MOUD engagement [26–30]. In this study, we use a CBPR method called “concept mapping” [31–33] to explore factors associated with MOUD engagement to develop a community-engaged response to increase MOUD uptake.

## Methods

### Overview and concept mapping

We conducted a pilot study called Welcome MAT that employed a mixed-methods concept mapping (CM) approach to identify facilitators and barriers to engagement in MOUD among service providers and persons with OUD in DC. CM includes obtaining a wide breadth of ideas about a topic from the general community (“brainstorming”), categorization and prioritization of those ideas (“sort and rate”), and community-based interpretation of the findings [31]. Although CM has been used to understand a variety of public health challenges and co-develop community-based programs [34–37], to our knowledge, CM has not been utilized to understand and develop strategies to improve MOUD engagement.

### Study population

The Welcome MAT study identified two primary populations of interest: persons with lived experience of OUD (referred to as consumers) and service providers. OUD is defined as a chronic disorder characterized by the chronic use of opioids that causes clinically significant distress or impairment. Consumers were individuals actively injecting or using opiates (<6 months) or who were entering or had been in recovery (not used/injected in ≥6 months) and may or may not have used MOUD services previously. “Service provider” was an intentionally broad term to include those whose work focuses on substance use issues, general health care providers who care for persons with OUD, and others from community-based organizations (CBOs) who serve the population impacted by OUD. Providers included primary care providers; emergency department providers; infectious disease specialists; clinical, behavioral health, or social support staff; and community health workers/peer recovery coaches.

We also formed a Welcome MAT community advisory board (CAB), whose members were consulted prior to the initiation of CM and later in the interpretation phase. The CAB consisted of individuals at various levels of involvement with persons with OUD in DC, including those with lived experience; people who provide harm reduction, treatment, and social services to persons with OUD; and providers and other professionals (e.g., DC Department of Behavioral Health, community-based organizations, peer recovery coaches, and academic researchers). These key stakeholders assisted in 1) establishing the current state of MOUD and other harm reduction programs in DC, 2) refining the research question, focus prompt, and CM methods, and 3) interpreting the study findings. CAB members were compensated for their time and expertise. The CAB included representatives from organizations that have been working with persons with OUD in the Washington, DC area for more than 30 years.

### Recruitment

The core activities of this study—brainstorming, sorting, and rating—were conducted from June 9, 2021-August 10, 2022. Several methods were used to recruit study participants to each activity. Flyers were distributed to CAB members, community-based organizations that work directly with consumers, and other local listservs (e.g., DC Center for AIDS Research, area MOUD provider lists, and primary care physicians). Additionally, the study was advertised via Craigslist, targeted social media posts, and institution-specific registries of potential study participants. As an incentive, we conducted a raffle of five $10 e-gift cards to participants who took part in the brainstorming phase using a random number generator. Consumers who participated in the sorting and rating phases received $50 gift cards.

### Concept mapping procedures

CM incorporates qualitative and quantitative analysis techniques through a modified four-step process [31]: 1) community brainstorming; 2) community sorting and rating; 3) multivariable statistical analyses; and 4) community interpretation and utilization of concept maps.

#### Step 1: Community brainstorming

To elicit an expansive list of statements, we asked consumers, interested community members (e.g., friends and family impacted by OUD), and professional stakeholders and clinicians who serve people with OUD to react and respond to the focus prompt, “What makes medication for opioid use disorder like buprenorphine (also known as Suboxone or Subutex) difficult to start or keep using?” While the prompt is inclusive of all “medication for opioid use disorder,” we mentioned buprenorphine due to the focus CAB members placed on it during our formative research and the greater flexibility in settings where it can be prescribed, as compared to methadone [38]. Additionally, we included the trade name of buprenorphine treatment to allow for greater community-based recognition of buprenorphine. All participants in the brainstorming phase provided between one and 10 open-ended responses to the question. We collected 98 statements, and after removing duplicate and unclear statements, 70 unique statements were finalized and examined in the sorting and rating stages of the CM process.

#### Step 2: Community sorting and rating

A total of 23 consumers and 34 providers participated in the sort and rate phase. Only consumers were involved in the in-person sorting phase. We printed each statement on an index card and asked individuals to sort similar or related statements into “piles” or categories. Participants then assigned descriptive titles/labels to each category based on the statements in their piles. Following the completion of sorting, participants rated each statement using a five-point Likert scale (with “1” indicating “not important” and “5” indicating “very important”), in response to the prompt: “Rate this item in terms of importance to accessing MAT.” Providers were only involved in the rating portion of this step. Through an online survey, they viewed the statement list and rated the items using the same prompt and scale.

Each group was asked to complete an optional survey recording demographic and other information including substance use history and prior experiences in/with treatment for consumers, and professional history and MOUD offerings in their respective workplaces for service providers. The surveys garnered responses from 21 of 23 consumers and 20 of 34 providers.

#### Step 3: Multivariate statistical analyses

Multivariate statistical analyses (i.e., multidimensional scaling and hierarchical cluster analysis) were conducted using CM software to create matrices and point estimates to analyze Sort and Rate data [39]. Initially, individual participant sort matrices were computed. Data from the individual sort matrices were used to create an aggregate matrix. Matrix values ranged from zero to the total number of sorters (i.e., consumer participants, n = 23). These values indicated how often participants sorted a statement with other like statements. The aggregate matrix was analyzed using multidimensional scaling (MDS) [39] to create a point map. Each point on the map represented a statement. The stress value for this study, or the measure of how well the point map reflected the similarity matrix and is interpretable [40], was 0.3451. Lower stress values are preferred (within the range of 0–1), and most CM projects yield results between 0.10 and 0.35 [40].

After MDS, hierarchical cluster analysis was used to analyze similarities in the data structure. The coordinates for each of the statements from the MDS analysis were used as data for the cluster analysis and aggregated to reveal overarching concepts [40]. The CM software suggested labels based on consumers’ pile titles (from Step 2 sorting) most like the final clusters shown. In some cases, we modified the cluster labels slightly for clarity and local context.

#### Step 4: Community interpretation and utilization

We presented preliminary results to the Welcome MAT CAB for community interpretation and utilization. During a focus group-like discussion, CAB participants were asked to 1) interpret the concept maps (ranging from six to eight clusters), 2) suggest modifications, and 3) decide how the results could be used to further develop the Welcome MAT program. The focus group was recorded and transcribed, and we used thematic coding and analysis to inform our interpretation and recommendations for program strategies.

All study activities were approved by the George Washington University Committee on Human Research, Institutional Review Board (NCR191958). All participants provided written consent.

## Results

### Sample description

In the sort and rate phase, the study sample consisted of 23 consumers (sort and rate) and 34 providers (rate-only). Most (95%) consumers were middle-aged Black men (53.8 years, SD = 10.9). More than half (52.4%) were Veterans, as indicated by their use of Veterans Administration insurance coverage. Prior experience using or injecting opioids was a pre-requisite to be in the study (whether in active addiction or recovery), and heroin (57.1%), marijuana (57.1%), and opioids (47.6%) were the most used substances among participants in the six months prior to sampling. Methadone and buprenorphine were the most common treatment options for those currently in treatment (23.8%) or with any history of treatment. All consumers who responded to the survey had been part of a drug treatment program at least once, with nearly one in five (19%) engaging in treatment “five or more” times. Consumers received treatment primarily through community clinics and hospital emergency departments. Within the prior 12-month period, 43% of consumers had experienced a heroin- or opioid-involved overdose.

Providers in the sample were 44.7 years old on average (SD = 12.5) and identified across different racial groups (45% non-Hispanic white, 35% Black, 15% Hispanic, 10% Asian, and 5% AI/AN). More than half (60%) identified as female. They reported working primarily in hospitals and Federally Qualified Health Centers. Their expertise also reached into other settings such as mental health centers, needle exchange programs, and pharmacies. Providers noted that buprenorphine and naltrexone were the most common treatment options offered in their clinics and workplaces, with one having offered methadone. At the time of sampling, only one in four were waivered to prescribe buprenorphine, which likely represented the mix of clinicians and non-clinical personnel in the study. Demographic characteristics for each group are shown in Table 1.

**Table 1:** Sample demographics.

Characteristic	Consumers (n = 21)	Providers (n = 20)
	n or mean (% or SD)	n or mean (% or SD)
*Age*	53.81 (10.90)	44.65 (12.49)
Race*		
American Indian or Alaska Native(AI/AN)	1 (4.76)	1 (5.00)
Black or African American	20 (95.24)	7 (35.00)
Hispanic, Latino, or Spanish Origin		3 (15.00)
Asian		2 (10.00)
White, Non-Hispanic	2 (9.52)	9 (45.00)
*Gender*		
Female	8 (38.10)	12 (60.00)
Male	12 (57.14)	6 (30.00)
Transgender	1 (4.76)	1 (5.00)
Other		1 (5.00)

Table 1 includes demographic characteristics of consumers and providers who participated in an optional survey during the study.

*Participants were asked to select all that apply, creating a percentage total > 100.

### Concept mapping results

#### Brainstorming, sorting, and rating

**Fig 1:**
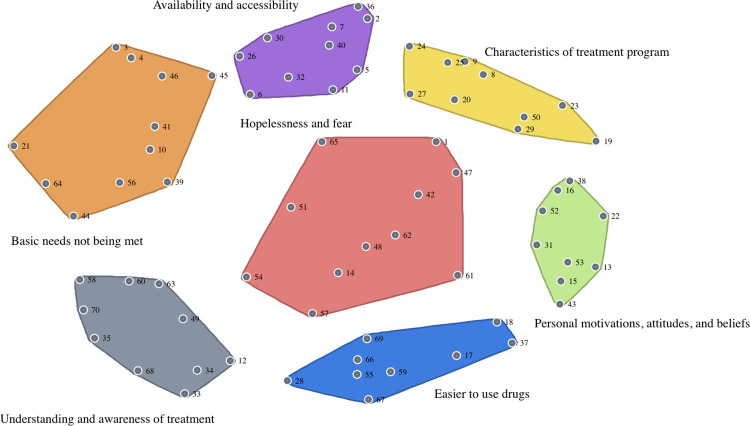
Cluster map. Each point on the cluster map corresponds to a participant-generated statement, listed in Table 2. The proximity of one point to another indicates that participants (i.e., consumers) sorted them together more frequently and found them to convey similar meanings.

The 70 unique statements generated during the brainstorming phase provided direct insight into the facilitators and barriers to engagement in MOUD. After reviewing the results with CAB members, our research team determined that seven clusters best encapsulated the data, outlining the following topics: 1) availability and accessibility, 2) hopelessness and fear, 3) basic needs not being met, 4) characteristics of treatment program, 5) understanding and awareness of treatment, 6) personal motivations, attitudes, and beliefs, and 7) easier to use drugs. Fig 1 displays the cluster map overlaid with the point map, and the corresponding statements grouped by cluster are shown in Table 2. The mean ratings of importance in Table 2 are inclusive of both consumer and provider rating data (n = 57). The subject matter of each cluster is explored below, in descending order from highest to lowest importance (i.e., mean cluster rating).

**Table 2:** Statements by cluster.

**Prompt: “What makes medication for opioid use disorder like buprenorphine (also known as Suboxone or Subutex) difficult to start or keep using?”**		**Rating of Importance, Mean (SD)**
**Availability and accessibility**		**4.05 (0.29)**
2	Not having access to technology to connect to programs	3.89
5	Being able to sign up for opioid use disorder treatment on the spot	4.39
6	Makes a big difference when it seems like my doctor really cares	4.44
7	Not having a phone makes it hard to stay connected to providers and fill prescriptions	4.36
11	Reduced ratio of medication-assisted treatment (MAT) providers to those seeking treatment	3.84
26	Allowing for larger take homes makes it easier	3.77
30	Treatment programs do not have enough hours on the weekends or nights	4.04
32	Monday to Friday, holidays off is not really how this works- need medication-assisted treatment (MAT) available all the time	4.21
36	Limited technology literacy. Hard to use tablet/phone when you are used to a desktop	3.47
40	Medications are not combined with behavioral counseling	4.11
**Hopelessness and fear**		**3.98 (0.28)**
1	Hard to get [medication assisted] treatment when basic needs are not being met	4.39
14	You will quit if you want to quit	3.82
42	Once people are doing bad they get depressed and they feel like they can just do whatever	3.43
47	Feel like I have no foundation (to even think about starting treatment)	4.26
48	People just don’t want to go through the sickness (so they are not willing to go through that to start buprenorphine)	3.87
51	People feel like it is too late for them, if they cannot get a home or if they got back from jail, just go back to the same place	4.06
54	It is difficult to start because you have to let yourself be in withdrawal for almost 3 days before you can take it unless you want to put yourself in the incident withdrawals which is hell on earth	3.94
57	The temptation to revert back to other opiate drugs like oxycodone and heroin	3.88
61	Majority of the people who have started using medication-assisted treatment (MAT) might fail to continue on the way due to stress	3.71
62	Lack of encouragement	4.00
65	People in remote places are unable to access medication-assisted treatment (MAT)	4.38
**Basic needs not being met**		**3.92 (0.35)**
3	Not having access to shelter/housing	4.60
4	Not having access to food	4.32
10	Mandatory drug testing promotes a return to dangerous fentanyl laced street drugs	3.43
21	Easy to get medication-assisted treatment (MAT)	4.23
39	Naltrexone requires full detoxification so initiating treatment among active users becomes difficult	3.91
41	Medication-assisted treatment is expensive	3.49
44	I have other medical issues that are more important	3.74
45	Case manager does not help	3.80
46	Medication assisted treatment does not offer vouchers for food or other things I need, I am always spending $10 to stay on someone’s couch	3.60
56	The inability to work certain jobs due to medication in system	3.81
64	Poverty. Many people who have opioid use disorder are not able to afford medication-assisted treatment	4.21
**Characteristics of treatment program**		**3.72 (0.20)**
8	Mandatory drug testing leads to decreased retention in opioid use disorder treatment programs	3.82
9	Mandatory drug testing in opioid use disorder treatment programs is stigmatizing	3.70
19	Forced because it is either jail or the treatment program so that is not really a choice	3.79
20	There are drugs at the treatment program	3.53
23	They do not really care at the treatment program, they are not trying to help for real	3.76
24	Services are not available at all times	4.15
25	There are legal barriers that fell during covid that have made it easier to get treatment	3.72
27	Urine tests	3.48
29	Drugs do not have urine tests or waiting times	3.43
50	People have bad mental health, they do not tackle that first	3.79
**Understanding and awareness of treatment**		**3.72 (0.34)**
12	Buprenorphine is not hard to get	3.44
33	Not enough promotion of Suboxone	4.02
34	Not enough awareness of Suboxone	4.06
35	Need more billboard campaigns or flyers of Suboxone	3.72
49	Suboxone being sold on the street	3.35
58	The fear of becoming physically and psychologically dependent on another substance	3.88
60	Some people are afraid of the medication side effects	4.00
63	Lack of awareness of medication-assisted treatment (MAT)	4.18
68	Opioids relaxes a lot, takes the pain away	3.13
70	Medication-assisted treatment is difficult to keep taking because it just makes your body feel terrible, it makes me feel like I want to be dead	3.41
**Personal motivations, attitudes, and beliefs**		**3.64 (0.41)**
13	You only do the treatment if you want to	4.11
15	You do not need the medicine to quit	3.46
16	The medicine is legal but otherwise medicine and drugs are the same	3.25
22	No one really wants treatment	2.91
31	Everyone has a moment where treatment is in the front of your mind and if you miss that moment, addicts are really good at making excuses not to make it today	3.89
38	Once you are complying with treatment, it creates dependency, which causes addiction to said medication	3.62
43	I am trying to start over, to get away from it before anything gets worse, like I could get HIV from some of these other guys	3.51
52	Cannot get people to do treatment before they are ready	4.33
53	People start young, becomes a way of life	3.69
**Easier to use drugs**		**3.44 (0.32)**
17	Methadone and buprenorphine make you nod just like drugs, so they are the same	2.91
18	You can get high off the prescription for free so people do that and also buy drugs and get high off that	3.29
28	Drugs are easier to get than Suboxone	3.4
37	You do not want to stop consuming the opioids	3.89
55	The social stigma of using buprenorphine/Suboxone	3.44
59	The long use of drugs like heroin makes it difficult to start the medication for opioid use disorder	3.87
66	I think because it is sometimes difficult to stick with and keep up as it is used multiple times a day	3.69
67	Buprenorphine/Suboxone does not completely help you either. It is a very hit or miss type of drug that can either really help you or be your downfall	3.48
69	Suboxone is expensive and addictive	3.00

The table includes 70 participant-generated statements, grouped by cluster. The values on the right represent the aggregate cluster ratings (bolded) and individual statement ratings using a five-point Likert scale (with “1” indicating “not important” and “5” indicating “very important”), in response to the prompt: “Rate this item in terms of importance to accessing MAT.”

#### Thematic clusters

*Availability and accessibility*. The 10 statements in the “availability and accessibility” cluster (shown in purple, Fig 1) allude to limitations in technology, hours of operation for treatment sites, timeliness of care, provider availability, and medication dosages offered. Out of the seven clusters, this cluster received the highest average rating of 4.05 (SD = 0.29). Statements highlighting connection, both emotionally and technologically, were rated higher in importance (e.g., “makes a big difference when my doctor really cares” and “not having a phone makes it hard to stay connected to providers”). Participants further emphasized the need for more accommodating hours, with one statement stating: “Monday to Friday, holidays off is not really how this works.”

*Hopelessness and fear*. The “hopelessness and fear” cluster (shown in red, Fig 1) had the second-highest average rating of 3.98 (SD = 0.28) and included 11 statements. Anticipated stress, lack of support, poor mental health, and it being “too late” all contributed to a sense of hopelessness, inhibiting the pursuit of treatment. Fear of withdrawal (and associated symptoms) is prominent, along with the fear of “failure” and return to use. Statement 65 (“People in remote places are unable to access medication-assisted treatment”) is somewhat of an outlier, but its location on the edge of the cluster and proximity to the “availability and accessibility” cluster suggests it is a bridging idea between the two.

*Basic needs not being met*. The “basic needs not being met” cluster (shown in orange, Fig 1) contained 11 statements and had a mean rating of 3.92 (SD = 0.35). Participants grouped statements about food and housing insecurity, poverty, the perceived cost of treatment, and competing health priorities. Without first addressing and satisfying these basic human needs and concerns, the idea of initiating and sustaining MOUD did not seem feasible for participants.

*Characteristics of treatment program*. There were 10 statements in the “characteristics of treatment program” cluster (shown in yellow, Fig 1) that addressed different aspects of the treatment environment. Mandatory drug testing (e.g., urine tests) was a recurring topic, carrying a negative connotation. Some participants found it stigmatizing and reduced retention rates. Wait times and service hours were also mentioned, echoing sentiments within the “availability and accessibility” cluster. Two statements drew attention to holistic health and a desire for providers to show genuine interest in and care for consumers’ mental health and overall well-being. The cluster had an average rating of 3.72 (SD = 0.2).

*Understanding and awareness of treatment*. The “understanding and awareness of treatment” cluster (shown in gray, Fig 1) included 10 statements and had an average rating of 3.72 (SD = 0.34). The cluster mentioned lack of awareness and promotion of MOUD in general, with one statement calling for increased campaign initiatives (e.g., billboards and flyers). Fear of the side effects of MOUD was also present in this cluster, as were perpetuated misconceptions of “becoming…dependent on another substance.” For some, the availability of buprenorphine on the street seems to have, in turn, raised awareness of these medications—although this street recognition may discourage treatment.

*Personal motivations*, *attitudes*, *and beliefs*. The nine statements that comprise “personal motivations, attitudes, and beliefs” (shown in green, Fig 1) were positioned closely together, and the cluster had an average rating of 3.64 (SD = 0.41). Most statements reflected various stages of an individual’s readiness to change, with one statement describing it as a fleeting choice: “Everyone has a moment where treatment is in the front of your mind and if you miss that moment, addicts are really good at making excuses not to make it today.” The cluster had undertones of internalized stigma and the concept of willpower, with engrained behaviors and a “way of life” being difficult to escape. The displayed attitudes towards treatment were not overwhelmingly positive, and participants expressed some doubt in MOUD’s efficacy in advancing recovery from OUD.

*Easier to use drugs*. The “easier to use drugs” cluster (shown in blue, Fig 1) had the lowest average rating at 3.44 (SD = 0.32). As the label suggests, its nine statements reiterated that it is easier for consumers to keep doing what they are already doing, as long as access to drugs remained less challenging than finding a treatment program that meets them where they are. The efficacy of MOUD was again questioned (e.g., “very hit or miss type of drug”) with some statements also identifying the misuse of such medications. Other noted barriers to MOUD in this cluster included cost and maintenance.

#### Pattern match

**Fig 2:**
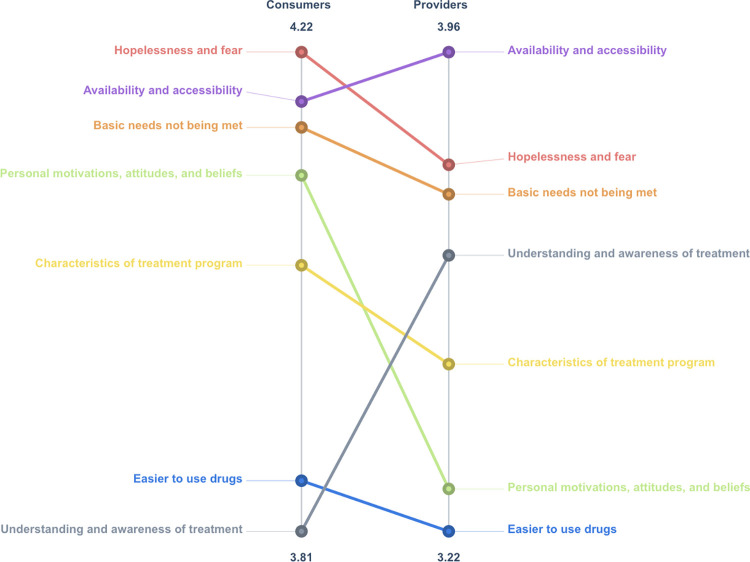
Pattern match. The pattern match shows the level of agreement between consumers and providers by separating the two groups’ rating data. The plotted points along the y-axes represent the aggregate cluster ratings using a 1–5 scale of importance (in response to “Rate this item in terms of importance to accessing MAT”). Both groups shared the same top three clusters (“hopelessness and fear,” “availability and accessibility,” and “basic needs not being met”), although the order of prioritization varied.

We next generated a pattern match to explore the relative importance of these seven themes and what level of agreement existed between consumers and providers (Fig 2). All participants generally found the subject matter of the statements to be important, rating them 3 and above on the scale (i.e., “moderately important,” “important,” or “very important”), but the pattern match illuminated discrepancies between the two groups’ rating data. The y-axis for consumers demonstrated that the average importance rating for the seven clusters was between 3.81 and 4.22. For providers, ratings were slightly lower, between 3.22 and 3.96. Both consumers and providers shared the same top three clusters (“hopelessness and fear,” “availability and accessibility,” and “basic needs not being met”). The order in which participants prioritized them was not a perfect match. Notable inversions in the figure include “personal motivations, attitudes, and beliefs” and “understanding and awareness of treatment.” Consumers placed personal motivation higher on the list of importance and awareness at the bottom, while providers gave more weight to awareness/promotion in improving access to and engagement in MOUD.

#### Community interpretation

These results were corroborated by CAB members during the interpretation phase of the CM process. Members underscored the importance of the “availability and accessibility” cluster, noting that existing service structures for MOUD provision do not always accommodate the lifestyles of the population. The inability to provide MOUD on the spot and get them engaged in the moment makes staying in contact with consumers and maintaining engagement in the treatment process very difficult as they compete with active addiction. Although not noted in the statements, other CAB members flagged transportation and lack of identification cards (IDs) as key structural challenges in accessing and delivering care. One of the organizations on the CAB has recognized this as a major barrier to receiving services and instituted a program to help individuals obtain IDs—an otherwise convoluted administrative process that can further reduce engagement and retention in MOUD.

Statements and concepts identified in the “basic needs” cluster aligned with accessibility; however, the identification of cost as a barrier to treatment did not resonate with CAB members. They noted most of the population served by their organizations have Medicaid and/or Medicare, and their treatment would be covered by insurance. However, this barrier may ultimately reflect misconceptions about what treatment services are available, free, or covered by insurance, or that because of their worry about basic needs, any additional costs (even if minimal) were not affordable. The clusters do not exist in silos; the impression that MOUD is “expensive” and individuals are “not able to afford [it]” relates to the knowledge gaps and lack of awareness identified in the “understanding and awareness of treatment” cluster.

In the interpretation of the “understanding and awareness of treatment” cluster, CAB members noted several discrepancies between the statements and the realities of treatment. They believed that people are aware of MOUD programs, clarifying that DC has “plenty of billboards” and other promotional initiatives. Instead, CAB members proposed that consumers might not understand all that MOUD encompasses, how the medications work, their long-term impact, and where to receive free care. They also recognized a need for such promotion and awareness-building to tackle stigma and misinformation.

The more “intangible areas” of the cluster map—as defined by a CAB member to include “hopelessness and fear” and “personal motivations, attitudes, and beliefs”—were notably more challenging to address. On the other hand, the “easier to use drugs” cluster resonated deeply with CAB members as a “tangible” fact in the community. From their perspective, the current environment makes it easier to obtain substances on the street than go through the process of finding a legitimate provider, enrolling in a treatment program, and following a medication protocol.

## Discussion

### Summary of findings

In this study, we identified seven themes based on participant self-reports of barriers to MOUD initiation and use. These themes encompassed various domains including withdrawal, fear, hopelessness, basic needs, and characteristics of treatment programs. Study findings showed that there is an urgent need for treatment programs to adapt certain strategies including extended clinical hours, expanded telemedicine and mobile services, an increased presence of peer recovery coaches, and improved coordination of services for consumers (e.g., housing assistance, food vouchers, and flexible take-home medication doses). Study findings also highlighted social-structural factors such as poverty and stigma, which contribute to addiction and challenge engagement in MOUD among our predominantly Black sample. These factors undergird Black men’s experiences with MOUD and care, and underscore the need for culturally-appropriate care to address them [4–6, 41]. Collectively, findings from this study demonstrated the complexity of opioid use and engagement in MOUD, and further support the importance of community-engaged approaches as a fundamental component of the public health response to the opioid crisis.

This study builds upon existing research on barriers to MOUD, specifically focusing on three key areas: accessibility of MOUD in various settings, including clinical spaces and consumer hotspots, lack of provider training and clinical support services for people seeking MOUD, and stigma surrounding MOUD and substance use [20–22, 42, 43]. Additionally, findings revealed that misinformation and disinformation about MOUD may be a growing problem among consumers, which not only hampers utilization of MOUD but also perpetuates negative attitudes towards treatment. For example, brainstorming statements included several misconceptions endorsed by consumers such as the belief that MOUD is ineffective, difficult to use, and mimics withdrawal symptoms. While many studies emphasize the need for improved access to MOUD and enhanced provider training [44–46], few reported on the consequences of misinformation and disinformation among consumers. Future research is needed to develop targeted messaging and marketing strategies for both providers and consumers. These strategies should be designed to dispel myths and misinformation about MOUD particularly related to efficacy, potential side effects, and costs.

Several clusters identified in this study described known social-structural determinants of OUD, such as poverty, food insecurity, and housing instability [4–6]. Given that this study was conducted during the height of COVID-19, legal barriers, limited access to technology, and the aforementioned social-structural determinants may have contributed as significant barriers to treatment and recovery [46, 47]. Although other studies indicated that people are more likely to sustain housing, employment, and recovery once they receive MOUD, the lack of stability and security in these areas greatly hinders treatment engagement [9–13]. Participant statements further confirmed the need for MOUD programs to be coupled with initiatives that provide housing stability and fulfill basic needs. For instance, the Housing First model or community-based case management interventions could be adapted to fit different settings, local needs, and treatment modalities [48, 49]. To improve nationwide access to care, it will be vital to continue to reduce barriers in treatment policies, reduce out-of-pocket expenses for insured individuals, and increase financial resources to support uninsured people throughout the treatment process. Moreover, although the provision of MOUD through telemedicine improved retention and health outcomes during the COVID-19 pandemic, inequities persist, with lower utilization of such services among people who are Black, AI/AN, and Asian or Pacific Islander [50]. Additional research is needed to identify best practices in delivering MOUD through telemedicine and to address broader barriers to MOUD among racial and ethnic minority populations.

Stigma towards people with OUD, which leads to experiences of isolation and discrimination, emerged as a key barrier in our study. There was a widespread belief reflected in the statements collected (e.g., statements 38 and 58) that MOUD simply replaces one drug addiction with another, which is consistent with other findings [21, 51]. Our CAB further described how these beliefs are commonly held within the broader community, exacerbating the stigmatization of people engaged in MOUD and ultimately serving as another barrier to treatment.

The CAB’s interpretation of the clusters and statements emphasized the critical need to develop innovative strategies that raise awareness about MOUD and the treatment process among the broader community including family members and employers. In addition, effective dissemination of accurate information could have a positive impact on consumers by increasing knowledge about the effectiveness of MOUD and fostering a supportive environment that encourages help-seeking behavior. For providers, there may be numerous opportunities to combat stigma towards populations that use drugs, including targeted education and training that avoids blaming individuals for opioid use [51]. The statements collected in this study further highlight the importance of addressing other aspects of patients’ lives, including their mental health, and connecting them to additional resources. This approach allows providers to demonstrate care for the consumer, recognize the consumer’s humanity, and establish a treatment environment that is more inclusive and destigmatizing.

### Strengths and limitations

Community engagement was a considerable strength of the study. By listening directly to consumers with lived experience of addiction and providers operating in treatment environments through each phase of the study, from brainstorming to interpretation, we were able to elicit meaningful and relevant findings and recommendations. Further, the most salient themes to emerge from the results aligned not only with CAB members’ experiences but also with existing literature on facilitators and barriers to MOUD. Another strength was the use of a mixed methods CM approach which contextualized, added depth, and resulted in an interpretable visualization of facilitators and barriers to MOUD.

The study had some limitations. The sorting phase for CM was complex, and the results did not fully capture the nuanced context within which this phase of the study occurred (i.e., conversations between participants and the research team regarding why statements were placed in certain piles over others). Nonetheless, CM has been successfully used with substance-using populations, and we followed best practices for data collection with this group [35, 52, 53], and the interpretation session with the CAB confirmed the validity of clusters. Another limitation was the sample size, which ideally would have been larger if not for the restrictions imposed by the COVID-19 pandemic. Nevertheless, the sample size was adequate for CM procedures [40]. Lastly, while the characteristics of our sample (e.g., majority Black men, veterans, engaged in drug treatment previously) reflect a portion of the consumer population of DC [2, 3], it does not reflect the population entirely and caution should be used when interpreting results with regards to sample bias. We acknowledge the rates of overdose and the makeup of persons with OUD in DC are unique, with higher rates than the national average and a population skewing older, which may limit the generalizability of study findings. Future studies should employ CM and CBPR to gain a better understanding of perceived facilitators and barriers to treatment among younger people with OUD, a growing demographic in the opioid crisis.

## Conclusion

OUD and overdose remain pervasive, cross-generational threats to health and well-being in the US. In our study, both consumers and providers highlighted various factors that hinder the initiation and maintenance of treatment for OUD. Recent efforts to create low-barrier access to MOUD, such as the expansion of telemedicine and telehealth services, peer-based support and navigation, shortened wait times, and pharmacy-based dispensing programs, demonstrate the effectiveness and urgent need for programs and interventions tailored to address community-specific needs [54–56]. CBPR can further support efforts to increase engagement of community members in developing and implementing MOUD services and create additional strategies to improve access to MOUD. Findings from this study have broader implications for public health research, policy, and practice. They emphasize the need to expand access to treatment, dismantle stigma associated with substance use and MOUD, and address the underlying circumstances that contribute to the profound sense of hopelessness and fear among persons with OUD. This paper importantly demonstrates the feasibility of using the concept mapping technique to assess the concordance and discordance between consumer and provider perceptions, and we hope that this technique may be used in other jurisdictions to enable collaboration and trust between these two main stakeholders. Increasing engagement in MOUD care is increasingly important in light of the continued epidemic of overdose fatalities both nationwide and in DC.
